# Associations between REM sleep EEG slowing and brain cholinergic denervation in aging and Mild Cognitive Impairment

**DOI:** 10.1038/s41380-026-03635-y

**Published:** 2026-05-12

**Authors:** Claire André, Marc-André Bédard, Véronique Daneault, Rebekah Wickens, Olga Fliaguine, Jean-Paul Soucy, Serge Gauthier, Dominique Lorrain, Célyne Bastien, Carol Hudon, Nicola Andrea Marchi, Jacques Montplaisir, Nadia Gosselin, Julie Carrier

**Affiliations:** 1 Centre for Advanced Research in Sleep Medicine, Hôpital du Sacré-Coeur de Montréal, Recherche CIUSSS NIM, Montréal, QC Canada; 2 Department of Psychology, Université de Montréal, Montréal, QC Canada; 3 NeuroQAM Research Center, Université du Québec à Montréal (UQAM), Montréal, QC Canada; 4 McConnell Brain Imaging Center, Montreal Neurological Institute (MNI), Montréal, QC Canada; 5 Emeritus Professor in Neurology and in Psychiatry; Co-lead, Dementia Education Program; McGill University, Montréal, QC Canada; 6 Research Centre on Aging, University Institute of Geriatrics of Sherbrooke, CIUSSS de l’Estrie-CHUS, Sherbrooke, QC Canada; 7 Department of Psychology, Université de Sherbrooke, Sherbrooke, QC Canada; 8 CERVO Brain Research Centre, Québec City, QC Canada; 9 School of Psychology, Université Laval, Québec City, QC Canada; 10 Department of Psychiatry, Université de Montréal, Montréal, QC Canada

**Keywords:** Prognostic markers, Neuroscience, Psychology

## Abstract

Cholinergic activity supports cortical activation during REM sleep, while other neurotransmitter systems are almost silent. Here, we tested the long-standing hypothesis that early cholinergic denervation may be associated with REM sleep EEG slowing in older adults. Twenty-four older participants without dementia (mean age: 71.29 ± 4.85 years; 58.33% women; 25% participants with amnestic Mild Cognitive Impairment) underwent a night of in-laboratory polysomnography, comprehensive neuropsychological evaluation, structural MRI and molecular PET imaging with [^18^F]-Fluoroethoxybenzovesamicol (FEOBV) to quantify brain cholinergic innervation. Voxel-wise multiple regressions assessed the associations between REM sleep characteristics (i.e., REM sleep percentage, relative theta power and EEG slowing ratios, defined as [delta + theta]/[alpha + beta] power) and FEOBV-PET standard uptake value ratio maps, controlling for sex. Given that FEOBV uptake was higher in women compared to men, we also performed exploratory sex-stratified analyses adjusted for age. Higher REM sleep EEG slowing over frontal (F3-F4), central (C3-C4), parietal (P3-P4), occipital (O1-O2) and temporal (T5-T6) derivations was significantly associated with cortical cholinergic denervation, notably in fronto-parietal areas and the medial temporal lobe. Sex-stratified analyses showed that higher REM sleep EEG slowing ratios were associated with cholinergic denervation mainly in medial temporal regions in women, and neocortical regions in men. These findings suggest that global REM sleep EEG slowing may represent a sensitive marker of cortical cholinergic denervation in older adults without dementia, and may constitute a promising marker for early diagnosis and disease-modifying interventions in Alzheimer's disease.

## Introduction

Rapid eye movement (REM) sleep is characterized by intense cortical activation, which is strongly driven by cholinergic activity, reaching its peak during this sleep stage [1–3]. The cholinergic neurons sustaining this cortical activation mainly arise from the basal forebrain, which project widely to the neocortex and limbic regions [4, 5]. Experimental evidence from animal models demonstrates that pharmacological blockade of cholinergic receptors, selective lesions of basal forebrain nuclei, or optogenetic suppression of cholinergic neurons profoundly disrupt REM sleep expression and induce slow-wave EEG activity [6–10]. Unlike wakefulness and non-REM sleep, REM sleep displays a unique neurochemical profile in which monoaminergic systems are largely inactive, while cholinergic tone predominates [3]. This marked cholinergic dependency makes REM sleep a privileged window for probing the integrity of the cholinergic system. Accordingly, cholinergic system alterations have been proposed to underlie REM sleep abnormalities in aging and neurodegenerative diseases, such as Alzheimer’s disease [11].

If aging is accompanied by a relatively modest reduction of REM sleep duration [12, 13], REM sleep is significantly altered in Alzheimer’s disease, a condition characterized by early and severe cholinergic loss [14–17]. In animal models of Alzheimer’s disease, REM sleep is often shortened and fragmented [18–20]. In humans, patients with Alzheimer’s disease exhibit a significant reduction of REM sleep duration, prolonged REM sleep latency and pathological slowing of EEG rhythms during REM sleep compared to cognitively healthy controls [21]. Such REM sleep EEG slowing is also already detectable in patients with amnestic Mild Cognitive Impairment, the prodromal stage of Alzheimer’s disease [22, 23]. Interestingly, promising results reported by our team show that reduced REM sleep duration is associated with lower basal forebrain volume in individuals with amnestic Mild Cognitive Impairment [24]. Still, direct in vivo evidence linking REM sleep characteristics to the integrity of the cholinergic system remains limited.

Over the last few years, several Positron Emission Tomography (PET) tracers have been developed to assess the integrity of the cholinergic system in vivo, targeting key components such as the acetylcholinesterase, acetylcholine receptors or the vesicular acetylcholine transporter (VAChT). Among them, ^18^F-fluroethoxybenzovesamicol (FEOBV) is the tracer showing the best affinity and specificity for the VAChT, present in presynaptic cholinergic terminals, thereby reliably reflecting presynaptic cholinergic density [25–28]. FEOBV-PET was found very sensitive to detect subtle cholinergic loss resulting from experimental lesions of the basal forebrain or pedunculopontine nuclei in rodents [29, 30]. In humans, recent FEOBV-PET studies have revealed significant cholinergic denervation in patients with Alzheimer’s disease [31, 32] or Mild Cognitive Impairment [33], mainly in the cingulate and temporo-parietal cortex, as well as in the frontal areas, with those reductions being directly correlated with the severity of basal forebrain atrophy [16, 31–33]. However, no study to date has directly assessed whether the loss of cholinergic terminals assessed using molecular imaging directly contributes to REM sleep alterations in older adults, including those at increased risk of Alzheimer’s disease. The recent development of FEOBV-PET, allowing for the specific in vivo quantification of presynaptic cholinergic terminals, now enables testing this long-standing hypothesis.

Here, we assessed whether cholinergic denervation, quantified using FEOBV-PET imaging, was associated with REM sleep EEG alterations. We tested this question in a sample composed of both cognitively unimpaired older adults and individuals with amnestic Mild Cognitive Impairment, to better capture potentially subtle cholinergic changes along the continuum spanning normal aging to preclinical and prodromal stages of Alzheimer’s disease. We hypothesized that REM sleep alterations – especially REM sleep EEG slowing, lower REM sleep proportion and/or lower REM sleep theta power – would be associated with cholinergic denervation, especially in fronto-temporal areas.

## Materials & methods

### Participants

Twenty-five participants were recruited between 2022 and 2024 from local memory and sleep clinics and the community in Montreal (Canada) and its surroundings. This sample size was chosen to align with prior FEOBV-PET studies in older populations [31–35]. Participants underwent a phone screening followed by an in-person interview to verify their potential eligibility, and were then invited for a comprehensive neuropsychological assessment and a full night in-laboratory polysomnography. Inclusion criteria were being 65 years old or above, fluent in French or English, having at least seven years of education and showing preserved autonomy in daily life. Exclusion criteria were the presence of dementia, the presence or history of major neurological disorders (e.g., epilepsy, traumatic head injury or encephalopathy), the presence of a psychiatric disorder diagnosed according to DSM-5 criteria (e.g., major depression or anxiety), a body mass index greater than 35 kg/m^2^, smoking, alcohol or drug abuse, heavy consumption of caffeinated beverages, uncontrolled diabetes or hypertension, presenting with sleep disorders (e.g., moderate-to-severe obstructive sleep apnea assessed with an apnea-hypopnea index ≥15, insomnia, REM sleep behaviour disorder assessed using video-polysomnography), the presence of cerebrovascular or pulmonary diseases (e.g., history of stroke, chronic obstructive pulmonary disease), the use of medication affecting sleep, cognition, mood or brain functioning (e.g., antidepressants, hypnotics, opioids), or any reason preventing from performing an MRI or PET scan (e.g., claustrophobia, metallic implants, annual nuclear exposure over 50 mSv).

All methods were conducted in accordance with the relevant guidelines and regulations. The protocol was approved by the ethics committee of the CIUSSS du Nord-de-l'île-de-Montréal (MP-32-2018-1537), and a written informed consent was obtained from each participant prior to the examinations, according to the declaration of Helsinki. Participants underwent a neuropsychological evaluation, MRI scan, FEOBV-PET scan and a full-night in-laboratory polysomnography in a mean interval of 5.75 ± 4.04 months.

### Neuropsychological testing and questionnaires

A comprehensive neuropsychological assessment was performed, encompassing global cognitive functioning (Montreal Cognitive Assessment; MoCA) and four specific cognitive domains: 1) learning and memory (Rey Auditory Verbal Learning test; Logical Memory from the Wechsler Memory Scale third edition; immediate and delayed recall of the Rey Complex Figure Test), 2) attention and executive functions (Digit Symbol Substitution Task-Coding of the WAIS-IV, Alpha-Span, Trail Making Test, Verbal fluency, Stroop Color-Word Test), 3) language (Boston Naming Test) and 4) visuo-spatial functions (Copy of the Rey Complex Figure Test, Lines orientation subtest of the Birmingham Object Recognition Battery). A clinical cognitive diagnosis was established by consensus between senior neuropsychologists, based on neuropsychological performance. As previously described [24], a cognitive domain was considered altered when participants presented with two or more Z-scores ≤1.5 standard deviation (SD) in a given cognitive domain, or if participants presented a MoCA score <26 accompanied by one Z-score ≤1.5 SD in at least two cognitive domains including memory. Following these criteria, participants for whom all cognitive domains were objectively preserved were classified as cognitively unimpaired *(n* = *18)*, and those with at least one impaired cognitive domain including memory were classified as having possible amnestic Mild Cognitive Impairment *(n* = *6)*. No participant presented with a non-amnestic Mild Cognitive Impairment profile.

In addition to cognitive tests, participants completed several questionnaires: the Geriatric Anxiety Inventory [36], the Geriatric Depression Scale [37], the Activities of Daily Living Scale - Mild Cognitive Impairment version [38], the Epworth Sleepiness Scale [39], the Vascular Burden Index [40] and the Cognitive Complaint Questionnaire (“Questionnaire de Plainte Cognitive”) [41].

### Polysomnography recording

We performed a full-night in-laboratory polysomnography using a Natus system (Brain Monitor, Trex and Embla NDx; bandpass 0.3-200 Hz, digitized at a sampling rate of 512 Hz), including 12 EEG electrodes placed on the scalp according to the international 10-20 system (F3, F4, C3, C4, T3, T4, T5, T6, P3, P4, O1, O2; referenced to A1-A2), an electrooculogram, electrocardiogram, and chin and leg electromyograms. We recorded respiration and oxygen saturation using oronasal canula and thermistors, thoraco-abdominal belts and a finger pulse oximeter. Sleep stages and respiratory events were scored in 30-second epochs by certified medical electrophysiology technologists at the Montreal site, according to standard international criteria of the American Academy of Sleep Medicine [42]. Standard sleep variables were extracted from the scoring of sleep recordings (i.e., sleep onset latency, total sleep time, sleep efficiency, wake after sleep onset, the proportion of time spent in each sleep stage, the number of awakenings per hour of sleep, and REM sleep onset latency). Regarding respiratory events, sleep apneas were defined as drops of ≥90% of airflow for a minimum of 10 seconds, and hypopneas were defined as a ≥ 30% reduction of airflow for a minimum of 10 seconds, followed by a cortical arousal and/or a ≥ 3% oxygen desaturation. Obstructive sleep apnea severity was estimated by the apnea-hypopnea index, corresponding to the number of apneas and hypopneas per hour of sleep. All participants presenting with an apnea-hypopnea index ≥15, indicative of untreated moderate-to-severe sleep apnea, were excluded. Lastly, periodic limb movement during sleep (PLMS) were also scored based on the 2012 American Academy of Sleep Medicine criteria [42], with leg movements occurring between -3.5 to +8.0 seconds around the end of an apnea or hypopnea not being scored as PLMS [43].

### Quantitative EEG analyses

#### Sleep EEG

All quantitative EEG analyses were performed using the Snooz toolbox (https://snooztoolbox.com). Prior to spectral analyses, artifacts were first automatically detected on NREM epochs (i.e., N2 and N3 epochs) [44], and then visually inspected by experienced sleep technologists. For REM sleep, sections of REM sleep free of eye movements or other artifacts were manually sampled from all REM sleep periods. Of note, one subject (woman aged 74 from the control group) did not have artifact-free REM sleep sections and was therefore not included in quantitative REM sleep EEG analyses. For the remaining 23 subjects, the mean duration of artifact-free REM sleep sections was 117.2 ± 12.8 seconds. Welch-like spectral power analyses were performed on 4-second epochs with a cosine tapering window (spectral resolution of 0.25 Hz). Absolute REM sleep spectral power was computed on all derivations on the following frequency bands: delta (0.5-4 Hz), theta (4-8 Hz), alpha (8-13 Hz), and beta (13-32 Hz). All absolute spectral power values were normalized to total spectral power, and log-transformed relative spectral power values were used in subsequent analyses. In addition, we computed REM sleep EEG slowing ratios using absolute power values, as follows: [(delta + theta)/(alpha + beta)]. As no hypothesis based on lateralization was made, we averaged spectral power and EEG slowing ratios bilaterally for all analyses, on frontal (F3-F4), central (C3-C4), parietal (P3-P4), temporal (T3-T4 and T5-T6) and occipital (O1-O2) derivations, for the main analyses. Topoplots of the REM sleep outcomes of interest (i.e., slowing ratios and theta power) are available in Supplementary Fig. 1.

For supplementary analyses, we also computed absolute NREM sleep spectral power on N2 and N3 epochs on the following frequency bands: delta (0.6-4 Hz), theta (4-8 Hz), alpha (8-12 Hz), sigma (12-16 Hz) and beta (16-32 Hz), on the same derivations.

#### Wakefulness EEG

For secondary analyses aiming at assessing the specificity and sensitivity of our main results, spectral power was also computed on resting-state wakefulness EEG recordings. For this purpose, a 10-minute resting state waking EEG recording was performed in the morning after the night of polysomnography, one hour after wake-up time to prevent sleep inertia. Participants were lying in bed with eyes closed, and were periodically asked to open their eyes to prevent drowsiness. We selected the maximum of artifact-free EEG sections encountered for each electrode (mean duration: 149.1 ± 80.2 seconds). We extracted spectral power from artifact-free sections and computed slowing ratios following the same procedure than for REM sleep sections.

### Neuroimaging examinations

A structural MRI (3 T GE Discovery MR750) and a FEOBV-PET scan (GE Discovery PET/CT 690) were completed on the same day for each participant at the PERFORM Centre of Concordia University (Montréal, Canada). A 3D T1-weighted MRI sequence was acquired with the following parameters: TR = 7.4 ms; TE = 3.06 ms; TI = 400 ms; matrix size = 256 × 256 × 176; FOV = 25.6 cm; voxel size = 1.0 mm isotropic; flip angle = 11°.

PET scan was performed three hours following a bolus intravenous injection of [^18^F]-Fluoroethoxybenzovesamicol, with a radioactivity ranging between 160 and 300 MBq. FEOBV was synthesized in the morning at the Cyclotron facility of the McConnell Brain Imaging Centre of the Montreal Neurological Institute (Montréal, Canada) and sent within an hour to the PERFORM Centre. A short low kV CT scan for attenuation correction was first performed. PET data acquisition was done in 3D list mode for a 30 min duration. PET images were reconstructed using an OSEM (Ordered Subset Expectation Maximization) algorithm with resolution recovery, into six frames of five minutes each. Reconstructed images were corrected for attenuation, scattering, random coincidences, decay and dead time. Frame-based motion correction was also performed if needed. As previously described [24], image preprocessing was conducted using the Computational Anatomy Toolbox version 12.7 (CAT12, Jena University Hospital, Germany; release 1653; www.neuro.uni-jena.de/cat/index.html) for SPM12 (Wellcome Trust Centre for Neuroimaging, London, UK; release 6906; https://www.fil.ion.ucl.ac.uk/spm), under MATLAB R2018b (MathWorks, Natick, MA, USA; https://www.mathworks.com). T1-weighted images were spatially normalized, segmented, registered to a reference template within the Montreal Neurological Institute (MNI) space was computed using the diffeomorphic anatomical registration through exponentiated lie algebra algorithm (DARTEL) [45], modulated and visually inspected for quality check. For supplementary analyses, we extracted the mean bilateral volume of basal forebrain nuclei (i.e., Ch123, Ch4 and total basal forebrain volume in mm^3^) from unsmoothed modulated gray matter maps, using regions of interest obtained from a probabilistic atlas created based on postmortem data [46], which were co-registered and resliced on the DARTEL template, as previously described [24]. The total intracranial volume (TIV) was calculated using the implemented TIV estimation module in CAT12.

For PET images, their six frames were time-averaged to create a single image. Similar linear transformation matrices from the MRI normalization were applied to the PET images to align them with the MRI. A Müller-Gärtner partial volume correction was applied to PET data using the PETPVE toolbox (http://www.fil.ion.ucl.ac.uk/spm/ext/#PETPVE12) [47, 48]. The DARTEL algorithm [45] was used to estimate the nonlinear deformation field, and these were applied to the PET scan, further aligning the PET images to the MNI152 template space. Standardized uptake value ratio (SUVR) maps were generated from spatially normalized PET images by using supratentorial white matter as the reference region, as previously used in several FEOBV-PET studies with similar populations because of the absence of specific tracer binding [31, 33, 49, 50]. Resulting images were smoothed using a Gaussian kernel with a full width at half-maximum of 8 mm. We excluded non-grey matter voxel from all analyses by applying a GM mask, created using the mean GM template of our population (thresholded at 0.2 and binarized).

### Statistical analyses

We assessed the normality and variance homogeneity of each variable using Shapiro-Wilk and Levene’s tests respectively, and non-normal variables were log-transformed prior to statistical analyses. In order to take into account potential confounders when exploring the associations between REM sleep and cholinergic innervation, we first verified whether FEOBV uptake was affected by demographics. Therefore, we performed (i) voxel-wise two-sample comparisons of FEOBV SUVR maps between men and women, as well as between cognitively unimpaired versus Mild Cognitive Impairment participants, and (ii) voxel-wise simple regressions between FEOBV SUVR maps and age and education, separately. We also assessed whether REM sleep outcomes (i.e., REM sleep percentage, theta power and EEG slowing ratios) were influenced by demographic variables (i.e., age, sex, education and cognitive status), by conducting simple regression analyses combined with an FDR correction for multiple comparisons for continuous variables (for age and education), and independent two-sample T-tests for categorical variables (for sex and cognitive status).

**Fig. 1:**
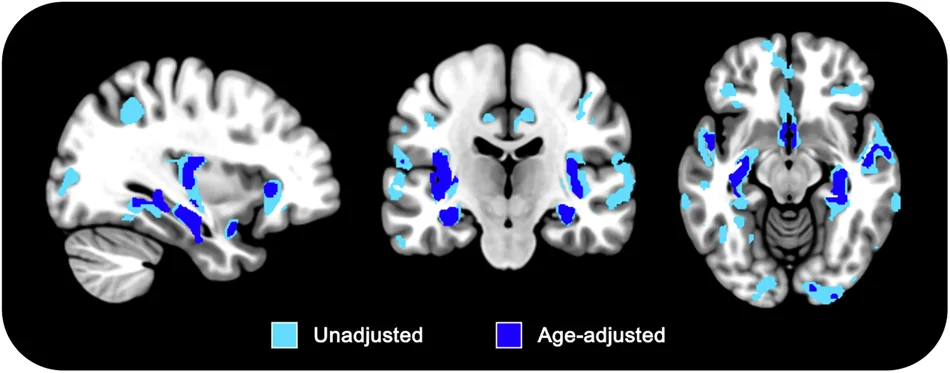
Sex differences in FEOBV uptake. Result of unadjusted (light blue) and age-adjusted (dark blue) two-sample comparisons of FEOBV uptake between women (*n* = 14) and men (*n* = 10) (Women>Men contrast). Significant clusters depict higher FEOBV uptake in women compared to men at the *P* < 0.005 uncorrected threshold, combined with a minimum cluster size of k = 100. Detailed clusters statistics and peak coordinates are displayed in Supplementary Table 1.

The main analyses investigated the associations between cholinergic denervation and REM sleep outcomes using whole-brain voxel-wise multiple regressions between FEOBV SUVR maps and REM sleep outcomes. For parsimony reasons, these analyses were only controlled for sex, as sex had a significantly impact on FEOBV uptake (see Fig. 1 and Results section). However, all results were then confirmed in fully adjusted models, controlled for age, sex and cognitive status, based on previous literature and as age was marginally associated with REM sleep percentage only (see Results section).

Several sets of supplementary analyses were then conducted to ensure the robustness and complement the main observations. First, we assessed whether the main voxel-wise results were replicated in sex-stratified samples, adjusting for age, as women were significantly older than men (see Table 1). Second, we ensured the specificity of the associations between cholinergic denervation and REM sleep by conducting voxel-wise regressions with NREM sleep or wakefulness EEG slowing ratios as independent variables. Lastly, we explored the associations between the volume of cholinergic basal forebrain nuclei and the outcomes of interest (i.e., REM sleep EEG slowing ratios, and the mean FEOBV-PET SUVr extracted from clusters significantly associated with REM sleep EEG slowing ratios), using multiple regressions controlled for sex and total intracranial volume (TIV). All voxel-wise neuroimaging analyses were carried out using Matlab R2022b and SPM12 (version 7771). The main analyses were considered significant at the *P* < *0.005* (uncorrected) threshold, combined with a cluster-level threshold of *P* < *0.05* corrected for family-wise error (FWE). More exploratory analyses in subsamples (i.e., sex-stratified analyses) are presented at a more permissive threshold of *P* < *0.005* (uncorrected), combined with a minimum cluster size of *k* = *100* voxels.

**Table 1:** Participants’ characteristics.

Variable	Whole sample (*n* = 24)		Women (*n* = 14)		Men (*n* = 10)		Between-group differences	
	Mean	SD	Mean	SD	Mean	SD	Statistic	*p*-value
Age: years	71.29	4.85	72.93	4.20	69.00	4.97	T = 2.10	**0.048**
Sex: number (%) women	14 (58.33%)		–		–		–	
Education: years	16.33	3.51	15.50	3.53	17.50	3.31	T = -1.41	0.174
Body mass index: kg/m^2^	25.26	4.33	25.44	5.04	25.00	3.30	T = 0.24	0.810
Montreal Cognitive Assessment: total score	26.75	2.66	27.50	2.14	25.70	3.06	T = 1.70	0.103
Possible aMCI: number (%)	6 (25%)		3 (50%)		3 (50%)		χ²=0.23	0.633
Vascular Burden Index: total score	0.54	0.88	0.43	0.85	0.70	0.95	U = 55.50	0.338
Cognitive Complaint Questionnaire: total score	2.38	2.26	3.07	2.62	1.40	1.17	T = 1.88	0.073
Activities of Daily Living questionnaire: total score	45.96	4.94	46.29	6.06	45.50	2.99	U = 98.00	0.102
Geriatric Depression Scale: total score	2.25	2.33	3.00	2.63	1.20	1.32	T = 1.98	0.060
Geriatric Anxiety Inventory: total score	3.83	4.82	5.43	5.57	1.60	2.22	T = 2.05	0.053
Epworth Sleepiness Scale: total score	4.21	3.67	4.43	4.43	3.90	2.42	U = 64.50	0.767
Sleep onset latency: minutes	23.33	28.43	15.82	12.79	33.85	40.24	U = 59.00	0.546
Total sleep time: minutes	344.54	57.44	344.71	58.77	344.30	58.67	T = 0.02	0.987
Wake after sleep onset: minutes	100.79	42.45	102.39	51.68	98.55	27.15	T = 0.21	0.832
Night-time awakenings: number	32.29	13.70	25.79	9.14	41.40	14.18	T = -3.29	**0.003**
Sleep efficiency: %	76.51	9.98	76.34	11.67	76.74	7.61	T = -0.09	0.926
NREM sleep stage 1: %	15.99	7.00	13.27	5.73	19.80	7.10	T = -2.49	**0.021**
NREM sleep stage 2: %	58.6	9.95	57.91	11.25	59.56	8.27	T = -0.39	0.699
NREM sleep stage 3: %	9.175	9.52	14.16	9.54	2.19	2.67	T = 3.84	**<0.001**
REM sleep: %	16.25	7.04	14.66	7.69	18.48	5.61	T = -1.33	0.196
Apnea-hypopnea index: number/hour	7.03	3.48	5.61	3.32	9.01	2.74	U = 29.50	**0.019**
Total sleep time with oxygen saturation <90%: minutes	1.84	2.99	1.99	3.31	1.68	2.63	U = 65.00	0.787
PLMS index: number/hour	25.84	26.89	25.87	19.44	32.66	27.57	T = -0.71	0.486

Demographic, clinical and sleep data of the 24 participants of the study. Statistical differences between men and women were assessed using two-sided Student’s t-tests for normally distributed continuous variables, Mann-Whitney tests for non-normally distributed continuous variables, and chi-squared tests for categorical variables, as appropriate.

Results in bold indicate significant differences at the p < 0.05 level.

%, percentage; *aMCI*, amnestic Mild Cognitive Impairment; *NREM*, non-rapid eye movement; *PLMS*, periodic limb movements during sleep; *REM*, rapid eye movement; *SD*, standard deviation.

## Results

### Participants

From the 25 subjects included in the present study, one participant did not complete the MRI scan due to a claustrophobia attack, and was excluded from the analytic sample. The final sample was composed of 24 subjects, including 14 women and 10 men, 18 cognitively unimpaired participants and six patients with a possible amnestic Mild Cognitive Impairment, with a mean age of 71.29 ± 4.85 years (range: 65-80 years) and a mean level of education of 16.31 ± 3.51 years (range: 10-23 years). Detailed demographic, clinical and sleep characteristics of the sample are presented in Table 1.

### Impact of demographics on REM sleep and FEOBV uptake

Women presented higher FEOBV uptake compared to men, mainly in the medial temporal lobe (e.g., hippocampus, parahippocampal gyrus, amygdala), basal forebrain, insula and temporal cortex (Fig. 1 and Supplementary Table 1). FEOBV uptake did not significantly differ between cognitively unimpaired participants and those with possible amnestic Mild Cognitive Impairment, and was not significantly associated with age or education levels. The main analyses were thus controlled for sex, and exploratory sex-stratified analyses were conducted.

Regarding the associations with REM sleep outcomes, only a marginal negative association was observed between lower REM sleep percentage and age *(r* = *-0.42, p*_*unc*._*=0.043)* (Supplementary Fig. 2), and relative theta power on O1-O2 derivations was higher in women (*t* = *2.43, p*_*unc*._*=0.024)* (Supplementary Fig. 3). There was no other significant association between REM sleep outcomes and age or education, and no other significant REM sleep differences between groups stratified by sex or cognitive status (Supplementary Fig. 2, Supplementary Fig. 3 and Supplementary Fig. 4).

### Associations between FEOBV uptake and REM sleep characteristics

#### Whole sample

**Fig. 2:**
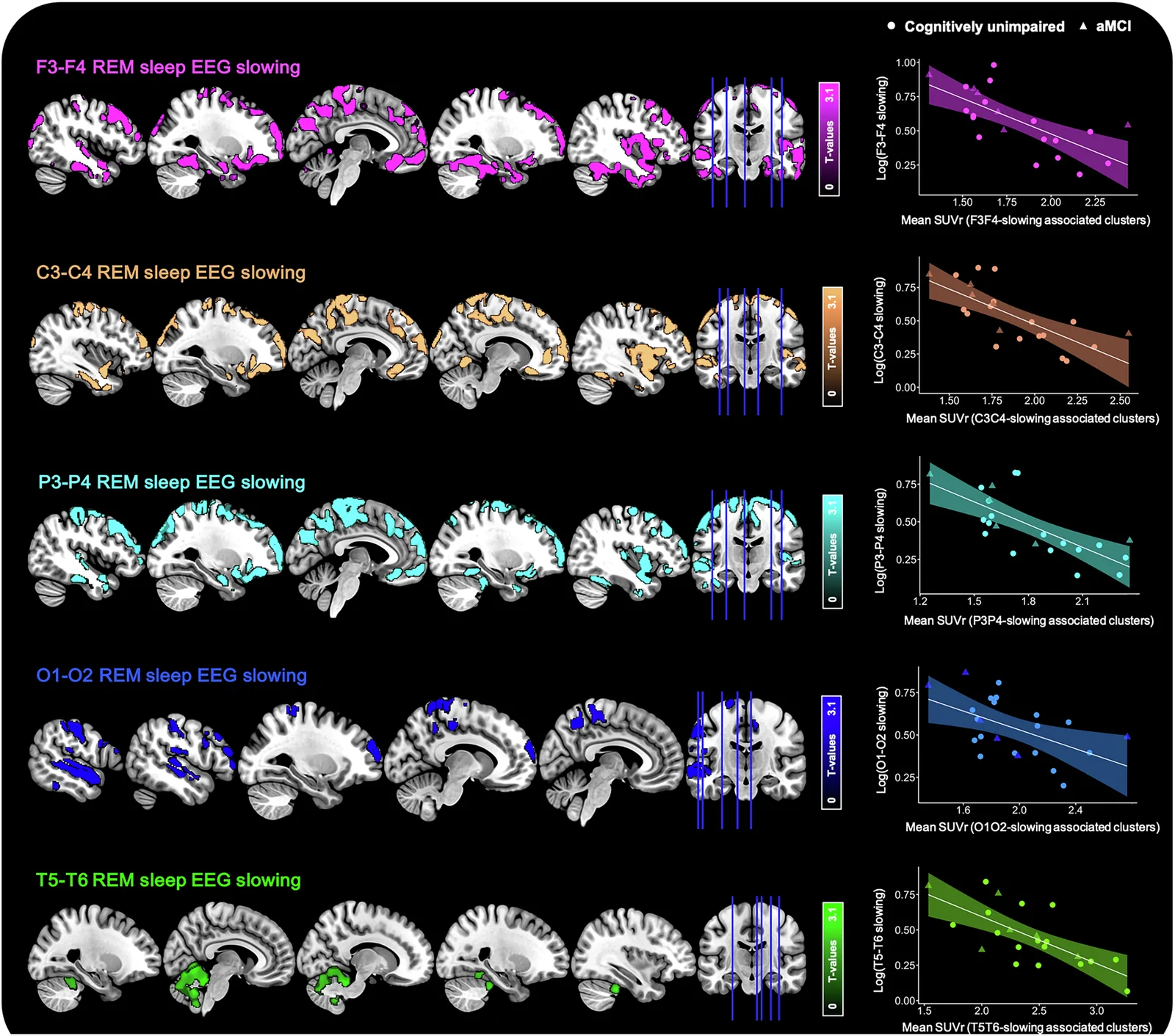
Associations between REM sleep EEG slowing ratios and FEOBV uptake. Negative voxel-wise regressions were performed between REM sleep EEG slowing ratio on F3-F4 (*n* = 22), C3-C4 (*n* = 22), P3-P4 (*n* = 23), O1-O2 (*n* = 23) and T5-T6 (*n* = 22) derivations and FEOBV SUVR maps corrected for partial volume effects, controlling for sex. Results are presented at the *P* < 0.005 uncorrected level, combined with a cluster-level FWE correction. Detailed clusters statistics and peak coordinates are available in Supplementary Table 2. Scatterplots show the associations between REM sleep EEG slowing ratio values, and the mean FEOBV signal extracted from the clusters significantly associated with REM sleep EEG slowing ratios in voxel-wise regressions.

We first investigated the associations between FEOBV uptake and REM sleep characteristics using voxel-wise multiple regression analyses, controlling for sex. Higher REM sleep EEG slowing over F3-F4, C3-C4, P3-P4 and O1-O2 derivations was significantly associated with a widespread reduction of FEOBV uptake, mainly in frontal, parietal (e.g., precuneus) and temporal areas, including the medial temporal lobe (Fig. 2 and Supplementary Table 2). Of note, the REM sleep EEG slowing ratio computed over T5-T6 electrodes was more marginally associated with FEOBV uptake in the cerebellum (Fig. 2 and Supplementary Table 2). These associations were replicated on individual electrodes (Supplementary Fig. 5), and still significant when adding age and cognitive status as covariates for F3-F4, C3-C4 and P3-P4 derivations (Supplementary Fig. 6)

However, no significant associations were found between FEOBV uptake and REM sleep proportion, relative theta power or REM sleep EEG slowing over T3-T4 electrodes.

In sensitivity and specificity analyses, we first verified whether cholinergic denervation was also associated with NREM sleep or resting-state wakefulness EEG slowing ratios, controlling for sex. NREM sleep EEG slowing ratios computed on the same derivations, and the wake EEG slowing ratios on P3-P4, C3-C4, T3-T4, T5-T6 and O1-O2 were not associated with FEOBV uptake at the permissive *P* < *0.005* uncorrected threshold, combined with a minimum cluster size of *k* = *100*. We only found a marginal negative association between the F3-F4 wake EEG slowing ratio and FEOBV uptake in the right superior temporal pole and left superior frontal gyrus, which did not survive a FWE cluster-level correction (Supplementary Table 3).

#### Sex-stratified analyses

As a significant sex-difference was observed in FEOBV uptake (Fig. 1), we verified whether the associations between FEOBV uptake and REM sleep EEG slowing ratios were replicated in sex-stratified subgroups. As women were slightly older than men (T = 2.10, p = 0.048; Table 1), age was added as a covariate in these analyses. Of note, the proportion of individuals with probable amnestic Mild Cognitive Impairment did not significantly differ between men and women (Table 1).

**Fig. 3:**
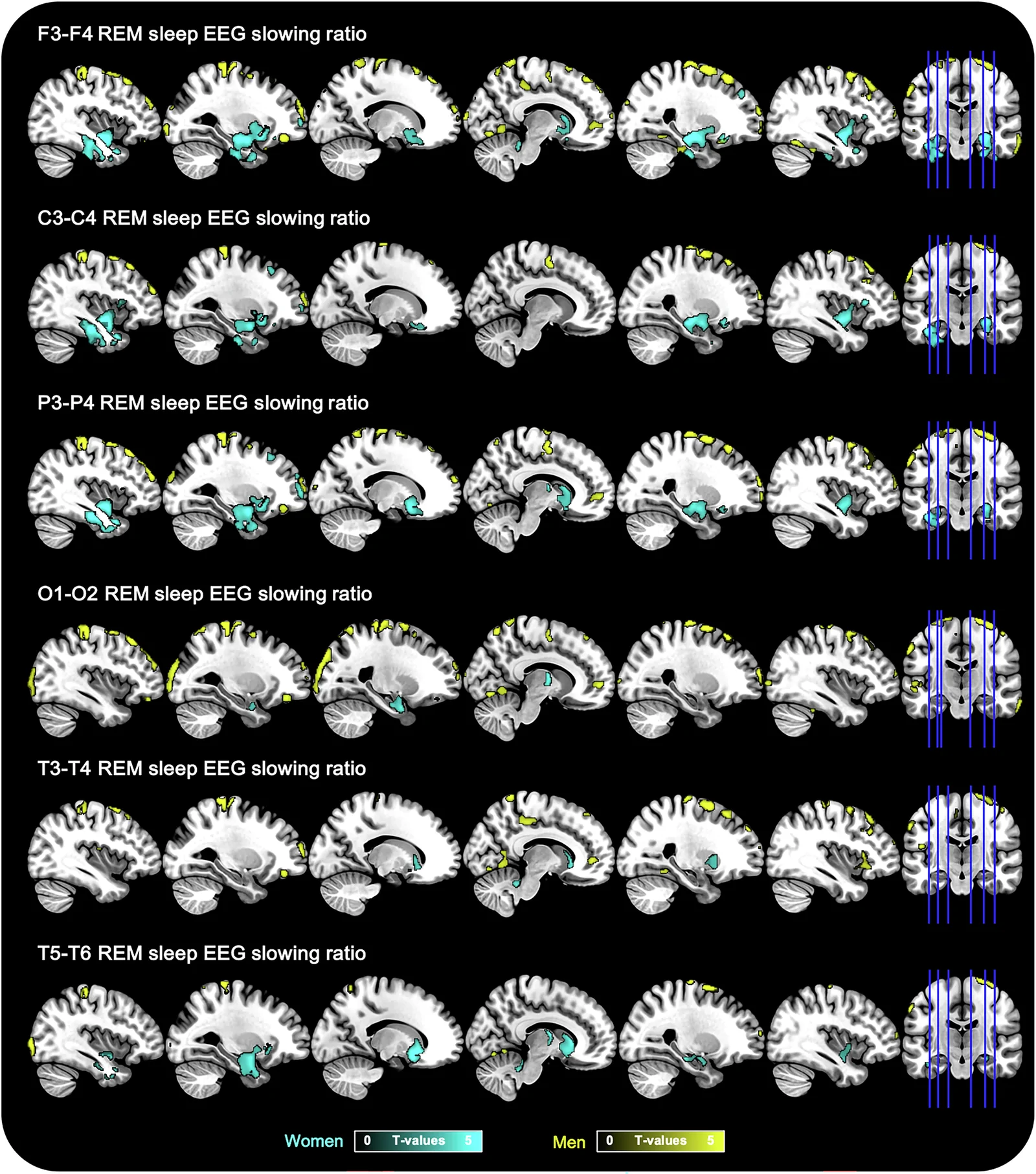
Differential associations between FEOBV uptake and REM sleep EEG slowing ratios in men and women. Sex-stratified negative voxel-wise regressions were performed between REM sleep EEG slowing ratio on F3-F4 (*n* = 12 women, *n* = 10 men), C3-C4 (*n* = 13 women, *n* = 9 men), P3-P4 (*n* = 13 women, *n* = 10 men), O1-O2 (*n* = 13 women, *n* = 10 men), T3-T4 (*n* = 13 women, *n* = 10 men), T5-T6 (*n* = 13 women, *n* = 9 men) derivations and FEOBV SUVR maps corrected for partial volume effects, controlling for age. Results are presented at the *P* < 0.005 uncorrected level, combined with a minimum cluster size of k = 100. Detailed clusters statistics and peak coordinates are available in Supplementary Table 4.

In women, we observed that higher REM sleep EEG slowing ratios over all derivations was mainly associated with lower FEOBV uptake in the medial temporal lobe (including the hippocampus and parahippocampal gyrus), amygdala, basal forebrain, insula, orbitofrontal and temporal cortex (Fig. 3 and Supplementary Table 4). In men, higher REM sleep EEG slowing ratio over all derivations was associated with lower FEOBV uptake mainly in the frontal, temporal and parietal neocortex (Fig. 3 and Supplementary Table 4).

### Associations with basal forebrain nuclei volume

To complement the main observations, we verified whether the volume of basal forebrain nuclei was significantly associated with REM sleep EEG slowing ratios or mean FEOBV uptake in regions significantly associated with REM sleep EEG slowing ratios, as identified in the main voxel-wise analyses. No significant associations surviving a FDR correction for multiple comparisons were found (Supplementary Table 5).

## Discussion

In this study, we provide the first direct in vivo evidence that brain cholinergic denervation is associated with REM sleep EEG slowing in older adults without dementia. This finding substantiates a long-standing hypothesis, suggesting that reduced cholinergic input to the cortex compromises the characteristics of EEG activity during REM sleep. This association was specific to REM sleep, as no significant relationship was found with NREM sleep or resting-state wakefulness EEG slowing ratios, underscoring the cholinergic dependency of REM sleep. Preliminary analyses also revealed sex-specific effects in the relationship between reduced cholinergic innervation and REM sleep EEG slowing, which require to be further clarified in future studies.

The fact that the slowing of EEG rhythms during REM sleep is sensitive to cholinergic denervation aligns with prior research emphasizing the key role of the cholinergic system in REM sleep physiology. Both wakefulness and REM sleep are characterized by an intense cortical activation with desynchronized EEG patterns, characterized by low-amplitude fast-frequency EEG rhythms. However, unlike wakefulness where cortical activation arises from the combined influence of multiple neurotransmitter systems (e.g., cholinergic, noradrenergic, serotoninergic, orexinergic) [51, 52], REM sleep critically depends on cholinergic inputs from brainstem and basal forebrain cholinergic nuclei, while other neurotransmitter systems are silenced during this sleep stage [1, 2, 51, 52]. Indeed, cholinergic neurons in the pedunculopontine (PPN) and laterodorsal tegmentum (LDT) are active during REM sleep, and are critical modulators of REM sleep physiology through their numerous projections to other structures [53–55]. They notably activate basal forebrain cholinergic neurons, which in turn amplify local neocortical activation and increase fast frequencies activity by releasing acetylcholine diffusely across the cortex [4, 5, 14, 54, 56]. Converging animal studies show that pharmacological, optogenetic and lesion-induced experimental disruption of cholinergic neurons reduces REM sleep and selectively promotes EEG delta activity [6–10]. Importantly, we here show that cholinergic denervation was specifically associated with EEG slowing during REM sleep, as no significant association was observed with EEG slowing ratios computed during NREM sleep or resting-state wakefulness, even at a more permissive statistical threshold. These results complement previous observations from a double-blind randomized clinical trial that increasing brain acetylcholine levels through the administration of anticholinesterase inhibitors such as donepezil reduce global REM sleep EEG slowing in Alzheimer’s disease patients [57]. Taken together, the dysfunction of cholinergic neurons in the brainstem and basal forebrain may underlie the decrease in fast frequencies power, and increase in low frequencies power, resulting in a global slowing of REM sleep EEG rhythms.

While cholinergic input from brainstem and basal forebrain nuclei is key for cortical activation during REM sleep, it is important to acknowledge that REM sleep regulation also depends on interactions with other modulatory systems, whose dysfunction may contribute to the observed associations between REM sleep EEG slowing and cholinergic denervation [54, 55, 58]. PPN/LDT neurons are known to project to other key regions contributing to REM sleep initiation, cortical activation and EEG desynchronization, or muscle atonia, such as the sublaterodorsal nucleus (SLD), thalamus and hypothalamus [54, 55]. Notably, melanin-concentrating hormone (MCH) neurons located in the lateral hypothalamus also play a pivotal role in REM sleep regulation by promoting EEG desynchronization and theta oscillations via cortical and hippocampal projections [59–62]. While replication in a larger sample of participants is necessary, higher global REM sleep EEG slowing may reflect the failure of a distributed network involved in cortical activation during REM sleep, and REM sleep quantitative EEG may constitute an early and specific marker of cholinergic denervation in older populations. Of note, we did not observe significant associations with REM sleep quantity. Given that our sample was mainly composed of cognitively unimpaired individuals, who usually show a relative preservation of REM sleep duration compared to patients with Mild Cognitive Impairment or Alzheimer’s disease [21, 63], it is possible that the reduction in REM sleep quantity may appear in individuals with more pronounced neuronal loss.

Cholinergic denervation may likely result from the early accumulation of Alzheimer’s disease pathology in cholinergic nuclei, as they are among the most vulnerable to the accumulation of abnormal proteins. Notably, basal forebrain neurons exhibit structural and functional properties making them especially vulnerable to early tau accumulation, including large and long-distance projecting axons, complex branching and high metabolic rates [64, 65]. Consistently, neuropathological studies have reported the presence of tau tangles in basal forebrain nuclei in the earliest stages of the disease [14, 66]. In addition to the basal forebrain, early tau deposition has also been documented in PPN/LDT cholinergic nuclei [67–69] – although neuronal loss seems less pronounced and may appear later than in the basal forebrain – and the lateral hypothalamus [66, 70, 71]. Therefore, the early accumulation of tau pathology in these nuclei, before cortical involvement and the onset of cognitive symptoms, is likely to influence both FEOBV-PET signal, and associated REM sleep cortical activation and EEG desynchronization, even in cognitively unimpaired older adults. In addition to tau, cholinergic neurons have also been shown to be sensitive to other pathological processes, notably including amyloid toxicity [72–76] and α-synuclein pathology [77, 78]. In particular, the basal forebrain is vulnerable to α-synuclein pathology [77, 78], and recent studies bring evidence of a reduction of FEOBV signal in DLB patients [49]. However, in our study, we did not include individuals with probable REM-sleep behavior disorder or polysomnographic evidence of REM sleep without atonia, which reduces the likelihood that our results may be driven by α-synuclein pathology. Similarly, even if mixed pathologies (e.g., vascular lesions, TDP-43) are frequent in the aging brain [79, 80], our participants exhibited low vascular risk factors, and TDP-43 accumulation in the basal forebrain does not seem to occur in the earliest stages of the disease [79]. Taken together, early tau accumulation especially in the basal forebrain is the most plausible pathological mechanism underlying cholinergic denervation even in individuals without cognitive impairment.

Of note, in our sample, basal forebrain volume was neither associated with FEOBV-PET signal, nor REM sleep EEG slowing ratio values. However, recent studies using FEOBV-PET have shown that patients with Alzheimer’s disease exhibit significant cholinergic denervation mainly in the temporo-parietal, medial prefrontal and cingulate cortices [31, 32], which correlates with the atrophy of Ch4 [16, 32]. A recent study showed similar associations between cholinergic denervation and basal forebrain atrophy in patients with Mild Cognitive Impairment [33]. One plausible explanation for our null finding is a limited sensitivity due to the small sample size, combined with the fact that our sample is mainly composed of cognitively unimpaired older adults. An alternative hypothesis would be that molecular dysfunction may emerge before macrostructural damage, with potential compensatory mechanisms at stake when individuals are still cognitively unimpaired. Future longitudinal studies will be essential to delineate the temporal sequence linking basal forebrain atrophy, cortical cholinergic denervation, and REM sleep alterations. Still, cholinergic denervation may have important clinical consequences. For example, basal forebrain atrophy has been associated to cognitive decline and the severity of memory and attentional deficits in Alzheimer’s disease and Mild Cognitive Impairment patients [81–83], and seems to predict conversion from Mild Cognitive Impairment to Alzheimer’s disease dementia [15, 84]. Here, no significant difference was observed in FEOBV-PET signal and REM sleep outcomes between cognitively unimpaired individuals and patients with amnestic Mild Cognitive Impairment. These observations need to be interpreted with caution as regards to statistical power, but it is possible that resilience to early AD pathology and associated neuronal loss (especially of vulnerable cholinergic neurons) in some cognitively unimpaired individuals may explain this absence of difference. Of importance, given the essential role of REM sleep in memory processing, synaptic plasticity and emotion regulation [85, 86], future studies will need to assess whether both cholinergic loss and associated REM sleep EEG slowing may impact cognition and psycho-affective health.

Lastly, our results provide preliminary insights into potential sex differences in the relationship between cholinergic denervation and REM sleep EEG slowing. In women, cholinergic innervation (mainly in the hippocampus, parahippocampal gyrus and amygdala) was higher than in men, and higher REM sleep EEG slowing was mainly associated with cholinergic denervation in the hippocampus, parahippocampal region, amygdala, basal forebrain, insula, orbitofrontal and temporal cortex. In men, global REM sleep EEG slowing was rather associated with cholinergic denervation in the fronto-temporo-parietal neocortex. Although these results should be interpreted with caution given the small sample size, they echo emerging evidence suggesting sex-specific patterns of vulnerability to Alzheimer’s disease pathology [87, 88]. Higher cholinergic innervation in women could reflect a compensatory upregulation of cholinergic innervation triggered by the early accumulation of Alzheimer’s disease pathology in cholinergic nuclei [89, 90]. Supporting this interpretation, evidence for an up-regulation of the activity of the cholinergic system in hippocampal regions has been suggested previously in individuals with Mild Cognitive Impairment, to compensate for an early neuronal loss in other medial temporal lobe subregions, such as the entorhinal cortex [89, 90]. The fact that this upregulation may be particularly evident in women could be due to higher medial temporal tau burden in the early stages of Alzheimer’s disease compared to men [87].

The main strengths of the present study include the in vivo assessment of cholinergic denervation using FEOBV-PET, combined with quantitative REM sleep EEG analyses, in a sample of older adults without dementia and moderate-to-severe obstructive sleep apnea. However, some limitations must be acknowledged. First, given the complexity of protocols including an FEOBV-PET procedure, the sample size of the present study is limited, even if it is in line with prior investigations in older populations [31–35]. Second, we did not have measures of Alzheimer’s disease biomarkers. We believe that this does not compromise our interpretations, as subcortical nuclei accumulate tau pathology at the earliest stages of the disease [66, 70, 91], before cortical involvement, such that a negative biomarker profile would not necessarily correspond to the absence of tau pathology in these regions. Still, we fully recognize that addressing whether tau pathology mediates the associations between cholinergic denervation and REM sleep EEG slowing represents a high-priority future direction. Future studies should replicate these observations in a larger sample of participants with well-characterized Alzheimer’s disease biomarkers (i.e., amyloid and tau pathology levels), and further clarify potential sex-specific effects. In addition, beyond cholinergic disturbances, it will be important to clarify the impact of the dysfunction of other neurotransmitter systems to REM sleep alterations in older adults, as our design does not allow to do so. Finally, our study was cross-sectional and longitudinal studies will be crucial to assess whether higher REM sleep EEG slowing ratios predict cognitive decline, the potential emergence of neuropsychiatric symptoms and conversion to dementia.

## Conclusion

Our findings provide the first direct evidence that higher global REM sleep EEG slowing is a sensitive and specific signature of cholinergic denervation in older adults. This work uncovers a key neurophysiological consequence of cholinergic degeneration, reinforcing the pivotal role of the cholinergic system in sustaining REM sleep cortical activity in humans. It highlights the importance of considering REM sleep alterations not only as a symptom of dementia, but as a possible window into early pathological changes. Clinically, quantitative REM sleep EEG may serve as a promising non-invasive marker for the early detection of neurodegenerative processes and a critical target for disease-modifying interventions in aging.

## Supplementary information

Supplemental Material

## Data Availability

Data used in the present study will be available from the corresponding author upon reasonable request.
