# Supplementary material for: Associations between REM sleep EEG slowing and brain cholinergic denervation in aging and Mild Cognitive Impairment
Source: Mol Psychiatry. 2026 May 12;31(10):5508–18. doi: 10.1038/s41380-026-03635-y (PMC13569431; doi:10.1038/s41380-026-03635-y)
Supplement: Supplementary file 1 [file 41380_2026_3635_MOESM1_ESM.docx]

**Supplementary Material**

**Associations between REM sleep EEG slowing and brain cholinergic denervation in aging and Mild Cognitive Impairment.**

Claire André, PhD, Marc-André Bédard, PhD, Véronique Daneault, PhD, Rebekah Wickens, MSc, Olga Fliaguine, MSc, Jean-Paul Soucy, MD PhD, Serge Gauthier, MD PhD, Dominique Lorrain, PhD, Célyne Bastien, PhD, Carol Hudon, PhD, Nicola Andrea Marchi, MD PhD, Jacques Montplaisir, MD PhD, Nadia Gosselin, PhD, Julie Carrier, PhD.

- **Supplementary Figure 1. Topoplots of REM sleep EEG spectral power measures of interest.**
- **Supplementary Table 1. Clusters showing higher cholinergic uptake in women compared to men.**
- **Supplementary Figure 2. Associations between REM sleep percentage and demographic variables.**
- **Supplementary Figure 3. Associations between REM sleep theta power and demographic variables.**
- **Supplementary Figure 4. Associations between REM sleep EEG slowing ratios and demographic variables.**
- **Supplementary Table 2. Clusters significantly associated with REM sleep EEG slowing ratios.**
- **Supplementary Figure 5. Associations between REM sleep EEG slowing ratios and FEOBV uptake on individual electrodes.**
- **Supplementary Figure 6. Fully adjusted associations between REM sleep EEG slowing ratio and cholinergic uptake.**
- **Supplementary Table 3. Association between the frontal resting-state wakefulness EEG slowing ratio and FEOBV-SUVr maps.**
- **Supplementary Table 4. Clusters significantly associated with REM sleep EEG slowing ratios in sex-stratified analyses.**
- **Supplementary Table 5. Associations between gray matter volume and REM sleep EEG slowing and FEOBV-PET signal.**

**Supplementary Figure 1. Topoplots of REM sleep EEG spectral power measures of interest.**

Topographic maps of REM sleep EEG slowing ratio values (*left*; ratio of the absolute power in slow/fast frequencies) and normalized theta power (4–8 Hz; *right*). For each electrode, values were averaged across participants and log-transformed. Abbreviations: Hz, Hertz; REM, rapid eye movement sleep.

**Supplementary Table 1. Clusters showing higher cholinergic uptake in women compared to men.**

| **Cluster label** | **k** | **T** | **Cluster-level p_FWE-corr_** | **MNI coordinates** | | |
| --- | --- | --- | --- | --- | --- | --- |
|  |  |  |  | **x** | **y** | **z** |
| **Unadjusted Two-sample analysis** | | | | | | |
| L hippocampus, middle temporal gyrus, superior temporal gyrus, supramarginal gyrus, Heschl gyrus, rolandic operculum, insula, superior temporal pole, putamen, basal forebrain, fusiform gyrus, lingual gyrus, parahippocampal gyrus, amygdala, thalamus, calcarine cortex, posterior cingulate cortex, precuneus | 7603 | 5.31 | **<0.001** | -32 | -18 | -12 |
| R hippocampus, parahippocampal gyrus, amygdala | 1691 | 4.99 | 0.18 | 28 | -33 | -3 |
| R lingual gyrus, calcarine cortex, superior occipital gyrus | 2577 | 4.86 | **0.048** | 14 | -92 | -8 |
| L middle temporal gyrus, inferior temporal gyrus, angular gyrus | 2768 | 4.28 | **0.036** | -45 | -74 | 20 |
| R supramarginal gyrus, inferior parietal gyrus, angular gyrus | 720 | 4.27 | 0.74 | 45 | -38 | 39 |
| R middle cingulate gyrus | 807 | 4.25 | 0.67 | 18 | -30 | 40 |
| L middle occipital gyrus | 477 | 4.25 | 0.91 | -27 | -82 | 8 |
| R insula, putamen, superior temporal gyrus, middle temporal gyrus, inferior temporal gyrus, supramarginal gyrus, Rolandic operculum, postcentral gyrus, precentral gyrus | 6777 | 4.24 | **<0.001** | 38 | -16 | 0 |
| L middle occipital gyrus, lingual gyrus, calcarine cortex | 1316 | 4.14 | 0.33 | -10 | -94 | 0 |
| L middle temporal gyrus, superior temporal gyrus, middle temporal gyrus, temporal pole | 1100 | 4.09 | 0.45 | -48 | -14 | -16 |
| L inferior frontal gyrus, middle frontal gyrus | 1376 | 4.04 | 0.30 | -52 | 24 | 8 |
| R middle temporal gyrus | 463 | 4.03 | 0.92 | 52 | -66 | -3 |
| R lingual gyrus, precuneus, calcarine cortex, cuneus. | 656 | 3.95 | 0.79 | 20 | -54 | 0 |
| B basal forebrain, olfactory cortex, gyrus rectus, superior frontal gyrus | 1124 | 3.86 | 0.44 | 2 | 9 | -10 |
| L inferior parietal gyrus | 633 | 3.82 | 0.81 | -33 | -50 | 44 |
| L precentral gyrus | 181 | 3.66 | 1.00 | -21 | -26 | 54 |
| L postcentral gyrus | 422 | 3.64 | 0.94 | -44 | -16 | 33 |
| R inferior frontal gyrus, middle frontal gyrus | 405 | 3.62 | 0.95 | 56 | 24 | 15 |
| L inferior frontal gyrus | 192 | 3.60 | 1.00 | -40 | 9 | 26 |
| B superior frontal gyrus, L anterior cingulate cortex | 725 | 3.53 | 0.74 | -14 | 62 | -9 |
| R lingual gyrus | 137 | 3.46 | 1.00 | 21 | -58 | -4 |
| R inferior frontal gyrus | 107 | 3.42 | 1.00 | 44 | 12 | 27 |
| R postcentral gyrus | 116 | 3.35 | 1.00 | 32 | -39 | 58 |
| L middle frontal gyrus, L inferior frontal gyrus | 209 | 3.27 | 1.00 | -46 | 32 | 27 |
| L inferior temporal gyrus | 111 | 3.16 | 1.00 | -57 | -18 | -28 |
| **Age-adjusted Two-sample analysis** | | | | | | |
| L hippocampus, parahippocampal gyrus, lingual gyrus, amygdala | 1627 | 4.63 | 0.20 | -32 | -18 | -14 |
| L insula, Heschl gyrus, Rolandic operculum | 766 | 4.52 | 0.70 | -38 | -15 | 15 |
| R hippocampus, parahippocampal gyrus | 856 | 4.43 | 0.63 | 32 | -30 | -10 |
| R lingual gyrus, inferior occipital gyrus | 368 | 4.12 | 0.96 | 14 | -92 | -8 |
| B basal forebrain, olfactory gyrus | 369 | 3.88 | 0.96 | 2 | 8 | -9 |
| R superior temporal gyrus, insula, Rolandic operculum. | 910 | 3.73 | 0.59 | 57 | -6 | 2 |
| R supramarginal gyrus | 149 | 3.66 | 1.00 | 58 | -27 | 32 |
| R superior temporal gyrus, middle temporal gyrus | 309 | 3.58 | 0.98 | 57 | -12 | -4 |
| L middle temporal gyrus | 467 | 3.48 | 0.92 | -56 | -56 | 8 |
| L superior temporal gyrus, Rolandic operculum, supramarginal gyrus | 506 | 3.45 | 0.90 | -57 | -34 | 22 |
| L superior temporal gyrus | 241 | 3.42 | 0.99 | -48 | 2 | -12 |
| L middle occipital gyrus, calcarine cortex | 101 | 3.39 | 1.00 | -10 | -94 | 0 |
| R superior temporal pole, amygdala, hippocampus | 138 | 3.32 | 1.00 | 28 | 3 | -22 |
| L inferior frontal gyrus, middle frontal gyrus, insula | 370 | 3.29 | 0.96 | -44 | 42 | -3 |

Results of two-sample voxel-wise comparisons showing higher cholinergic uptake in women (n=14) compared to men (n=10), at the P < 0.005 uncorrected level combined with a minimum cluster size of k=100. Clusters surviving a cluster-level FWE correction are highlighted in bold. For each cluster, upper coordinates correspond to the statistical peak of the cluster.

Abbreviations: FWE, family wise error; L, left; MNI, Montreal Neurological Institute; Nb, number; R, right.

**Supplementary Figure 2. Associations between REM sleep percentage and demographic variables.**

Associations between REM sleep percentage and demographic variables. Between-group differences in REM sleep outcomes according to sex or cognitive status were assessed using Student T-tests, and associations with age and education are assessed using Pearson’s correlations. The statistical threshold for significance was set to *P < 0.05*.

Abbreviations: Hz, Hertz; REM, rapid eye movement; TST, total sleep time.

**Supplementary Figure 3. Associations between REM sleep theta power and demographic variables.**

Associations between REM sleep theta power and demographic variables. Between-group differences in REM sleep outcomes according to sex or cognitive status were assessed using Student T-tests, and associations with age and education are assessed using Pearson’s correlations. The statistical threshold for significance was set to *P < 0.05*.

Abbreviations: Hz, Hertz; REM, rapid eye movement; TST, total sleep time.

**Supplementary Figure 4. Associations between REM sleep EEG slowing ratios and demographic variables.**

Associations between REM sleep EEG slowing ratios and demographic variables. Between-group differences in REM sleep outcomes according to sex or cognitive status were assessed using Student T-tests, and associations with age and education are assessed using Pearson’s correlations. The statistical threshold for significance was set to *P < 0.05*.

Abbreviations: Hz, Hertz; REM, rapid eye movement; TST, total sleep time.

**Supplementary Table 2. Clusters significantly associated with REM sleep EEG slowing ratios.**

| **Cluster label** | **k** | **T-value** | **p_FWE-corrected_** | **MNI coordinates** | | |
| --- | --- | --- | --- | --- | --- | --- |
|  |  |  |  | **x** | **y** | **z** |
| **F3-F4 REM sleep EEG slowing ratio (n=22)** | | | | | | |
| B vermis, R lingual gyrus, B fusiform gyrus, B middle temporal gyrus, B superior temporal gyrus, B inferior temporal gyrus, B temporal pole, R parahippocampal gyrus, B hippocampus, B amygdala, B insula, R postcentral gyrus, R precentral gyrus, R supplementary motor area, R superior frontal gyrus, B middle frontal gyrus, R inferior frontal gyrus pars orbitalis, B superior frontal gyrus-medial orbital, R inferior frontal gyrus-opercular part, R inferior frontal gyrus-triangular part, B anterior cingulate cortex, B rectus gyrus, B medial orbital gyrus, B posterior orbital gyrus, L anterior orbital gyrus. | 38622 | 5.58 | <0.001 | 6 | -58 | -6 |
| B cuneus, L precuneus, L angular gyrus, L middle and superior occipital gyrus, L calcarine cortex. | 2667 | 5.51 | 0.014 | -6 | -78 | 28 |
| B precuneus, R superior parietal gyrus, L inferior parietal gyrus, B middle cingulate gyrus, B postcentral gyrus, L precentral gyrus, B paracentral lobule, B supplementary motor area, L superior frontal gyrus, L middle frontal gyrus. | 8201 | 4.84 | <0.001 | -14 | -46 | 72 |
| L superior frontal gyrus, L middle frontal gyrus, L inferior frontal gyrus-triangular part. | 5451 | 4.48 | <0.001 | -2 | 45 | 45 |
| **C3-C4 REM sleep EEG slowing ratio (n=22)** | | | | | | |
| B inferior frontal gyrus, B superior frontal gyrus, B middle frontal gyrus, B rectus gyrus, R frontal medial orbital gyrus, B insula, B fusiform gyrus, B inferior temporal gyrus, B middle temporal gyrus, B superior temporal gyrus, R superior temporal pole, R Heschl gyrus, R Rolandic operculum, B middle cingulum gyrus, B precuneus, L inferior parietal gyrus, R superior parietal gyrus, B postcentral gyrus, L supramarginal gyrus, B precentral gyrus, R superior occipital gyrus, R supplementary motor area, B paracentral lobule. | 32757 | 5.63 | <0.001 | 39 | 20 | 8 |
| B cuneus, L superior parietal gyrus, L inferior parietal gyrus, L calcarine cortex, B superior occipital gyrus. | 3081 | 5.33 | 0.009 | -6 | -78 | 28 |
| L middle frontal gyrus, L superior frontal gyrus, L inferior frontal gyrus, L anterior cingulate cortex, L precentral gyrus. | 3222 | 4.68 | 0.007 | -45 | 22 | 40 |
| L superior frontal gyrus, L middle frontal gyrus, L inferior frontal gyrus. | 2641 | 4.56 | 0.020 | -14 | 62 | 21 |
| B inferior frontal gyrus, B superior frontal gyrus, B middle frontal gyrus, B rectus gyrus, R frontal medial orbital gyrus, B insula, B fusiform gyrus, B inferior temporal gyrus, B middle temporal gyrus, B superior temporal gyrus, R superior temporal pole, R Heschl gyrus, R Rolandic operculum, B middle cingulum gyrus, B precuneus, L inferior parietal gyrus, R superior parietal gyrus, B postcentral gyrus, L supramarginal gyrus, B precentral gyrus, R superior occipital gyrus, R supplementary motor area, B paracentral lobule. | 32757 | 5.63 | <0.001 | 39 | 20 | 8 |
| **P3-P4 REM sleep EEG slowing ratio (n=23)** | | | | | | |
| B cuneus, B precuneus, B superior occipital gyrus, L middle occipital gyrus, B fusiform gyrus, B postcentral gyrus, B superior parietal gyrus, L inferior parietal gyrus, B precentral, B superior frontal gyrus, B middle frontal gyrus, B inferior frontal gyrus-opercular part, B inferior frontal gyrus-triangular part, B inferior frontal gyrus pars orbitalis, R Rolandic operculum, B supplementary motor area, B olfactory cortex, B superior frontal gyrus-medial and medial orbital parts, B rectus gyrus, B orbital gyrus (anterior, medial and posterior parts), R anterior cingulate cortex, B insula, B middle cingulate gyrus, L hippocampus, L amygdala, B paracentral lobule, L putamen, R Heschl gyrus, B superior temporal gyrus, B temporal pole, B middle temporal gyrus, B inferior temporal gyrus, B cerebellar hemispheres. | 51051 | 4.25 | <0.001 | -6 | -78 | 28 |
| B vermis, R fusiform gyrus, R lingual gyrus, R parahippocampal gyrus. | 2170 | 3.72 | 0.039 | 4 | -60 | -6 |
| **O1-O2 REM sleep EEG slowing ratio (n=23)** | | | | | | |
| L middle temporal gyrus, L superior temporal gyrus, L inferior temporal gyrus, L Rolandic operculum, L supramarginal gyrus. | 3887 | 5.23 | 0.003 | -56 | -24 | -2 |
| L middle frontal gyrus, L superior frontal gyrus, L inferior frontal gyrus, L precentral gyrus. | 2846 | 4.75 | 0.015 | -28 | 54 | 24 |
| L inferior parietal gyrus, L precentral gyrus, L postcentral gyrus, L supramarginal gyrus, B superior parietal gyrus, B middle cingulate gyrus, B precuneus, R supplementary motor area, B paracentral lobule. | 4891 | 3.97 | 0.001 | -52 | -27 | 48 |
| **T5-T6 REM sleep EEG slowing ratio (n=22)** | | | | | | |
| B cerebellar hemispheres, B vermis, R fusiform gyrus, R lingual gyrus | 5253 | 5.84 | <0.001 | 9 | -45 | -26 |

Whole-brain voxel-wise negative multiple regression analyses were performed between REM sleep EEG slowing ratios on frontal (averaged F3-F4) and parietal (averaged P3-P4) derivations, and FEOBV SUVR maps. Results are presented at the *P < 0.005* (uncorrected) level combined with a FWE cluster-level correction, controlling for sex. For each cluster, upper coordinates correspond to the statistical peak of the cluster.

Abbreviations: B, bilateral; FWE, family wise error; L, left; MNI, Montreal Neurological Institute; R, right; REM, rapid eye movement.

**Supplementary Figure 5. Associations between REM sleep EEG slowing ratios and FEOBV uptake on individual electrodes.**

Figure presenting significant negative voxel-wise regressions performed between REM sleep EEG slowing ratio computed on each electrode and FEOBV SUVR maps corrected for partial volume effects, controlling for sex. Results are presented at the *P<0.005* uncorrected level, combined with a cluster-level FWE correction.

**Supplementary Figure 6. Fully adjusted associations between REM sleep EEG slowing ratio and cholinergic uptake.**

Significant whole-brain negative voxel-wise regressions between REM sleep EEG slowing ratio on F3-F4 (*n=22*), P3-P4 (*n=23*) and C3-C4 (*n=22*) derivations and FEOBV-SUVR maps corrected for partial volume effects, controlling for age, sex and cognitive status. Results are presented at the *P<0.005* uncorrected level, combined with a cluster-level FWE correction.

Abbreviations: FEOBV, [^18^F]-Fluoroethoxybenzovesamicol; FWE, family wise error; SUVR, standard uptake value ratio, REM, rapid eye movement.

**Supplementary Table 3. Association between the frontal resting-state wakefulness EEG slowing ratio and FEOBV-SUVr maps.**

| **Cluster label** | **k** | **T-value** | **p_FWE-corrected_** | **MNI coordinates** | | |
| --- | --- | --- | --- | --- | --- | --- |
|  |  |  |  | **x** | **y** | **z** |
| Right superior temporal pole | 107 | 4.38 | 1.000 | 36 | 9 | -27 |
| Left superior frontal gyrus | 193 | 3.98 | 0.997 | -26 | 15 | 51 |
| Left superior frontal gyrus | 137 | 3.79 | 0.999 | -26 | 39 | 44 |

Whole-brain voxel-wise negative multiple regression between the resting-state wakefulness EEG slowing ratio computed on frontal derivations (averaged F3-F4, *n=18*) and FEOBV-SUVr maps, controlling for sex. Results are presented at the *P < 0.005* (uncorrected) level, combined with a minimum cluster size of *k=100*.

Abbreviations: FEOBV, [^18^F]-Fluoroethoxybenzovesamicol; FWE, family wise error; SUVr, standard uptake value ratio; REM, rapid eye movement.

**Supplementary Table 4. Clusters significantly associated with REM sleep EEG slowing ratios in sex-stratified analyses.**

| **Cluster label** | **k** | **T-value** | **p_FWE-corrected_** | **MNI coordinates** | | |
| --- | --- | --- | --- | --- | --- | --- |
|  |  |  |  | **x** | **y** | **z** |
| **F3-F4 REM sleep EEG slowing ratio** | | | | | | |
| ***Women*** | | | | | | |
| Vermis | 220 | 7.80 | 0.995 | 6 | -40 | -21 |
| R hippocampus, parahippocampal gyrus, putamen, amygdala, basal forebrain, nucleus accumbens, gyrus rectus, medial orbital gyrus | 3651 | 1.36 | **0.003** | 27 | -20 | -15 |
| L insula, superior temporal gyrus, inferior temporal gyrus, middle temporal gyrus, amygdala, hippocampus, putamen, fusiform gyrus, basal forebrain, gyrus rectus, medial orbital gyrus | 5307 | 6.88 | **<0.001** | -39 | -9 | -15 |
| L anterior cingulate cortex | 142 | 4.55 | 0.999 | -2 | 38 | 6 |
| L superior frontal gyrus | 133 | 4.41 | 1.000 | -24 | 58 | 4 |
| R superior frontal gyrus | 101 | 4.16 | 1.000 | 22 | 44 | 38 |
| ***Men*** | | | | | | |
| B lingual gyrus | 582 | 8.98 | 0.296 | 6 | -62 | -2 |
| L precentral gyrus, supplementary motor area, precuneus, paracentral lobule, superior parietal gyrus, postcentral gyrus | 458 | 8.80 | 0.511 | -22 | -22 | 72 |
| L postcentral gyrus, precentral gyrus, paracentral lobule | 624 | 8.27 | 0.243 | -33 | -30 | 58 |
| R superior frontal gyrus, precentral gyrus | 2140 | 7.84 | **<0.001** | 27 | 27 | 56 |
| R middle frontal gyrus | 322 | 7.12 | 0.816 | 42 | 8 | 56 |
| L superior frontal gyrus | 293 | 7.06 | 0.873 | -14 | 38 | 54 |
| R precentral gyrus, postcentral gyrus | 180 | 6.83 | 0.992 | 63 | -2 | 27 |
| R superior parietal gyrus, precuneus, paracentral lobule | 818 | 6.74 | 0.097 | 15 | -63 | 68 |
| L postcentral gyrus, supramarginal gyrus | 294 | 6.63 | 0.871 | -57 | -14 | 46 |
| L supplementary motor area, paracentral lobule, middle cingulate cortex | 182 | 6.53 | 0.991 | -10 | -22 | 50 |
| R superior frontal gyrus | 169 | 6.23 | 0.995 | 27 | 62 | 15 |
| L orbitofrontal cortex | 221 | 6.02 | 0.969 | -27 | 39 | -14 |
| L middle frontal gyrus | 242 | 5.81 | 0.949 | -50 | 21 | 38 |
| L middle temporal gyrus, angular gyrus, superior temporal gyrus | 754 | 5.71 | 0.132 | -64 | -32 | 3 |
| R superior frontal gyrus | 233 | 5.68 | 0.958 | 20 | 66 | -8 |
| R superior frontal gyrus | 191 | 5.62 | 0.988 | 8 | 56 | -2 |
| L precuneus, superior parietal gyrus | 243 | 5.59 | 0.948 | -33 | 24 | 51 |
| L precuneus, superior parietal gyrus, paracentral lobule | 447 | 5.59 | 0.534 | -4 | -42 | 74 |
| L supplementary motor area, L superior frontal gyrus | 113 | 5.56 | 1.000 | -12 | 22 | 62 |
| R supplementary motor area | 102 | 5.45 | 1.000 | 6 | 18 | 66 |
| R fusiform gyrus | 237 | 5.43 | 0.954 | 30 | -33 | -26 |
| L middle occipital gyrus, inferior occipital gyrus | 152 | 5.42 | 0.998 | -28 | -94 | -2 |
| L precentral gyrus, middle frontal gyrus, postcentral gyrus | 359 | 5.30 | 0.734 | -42 | -6 | 62 |
| R supplementary motor area | 123 | 5.26 | 1.000 | 8 | -6 | 57 |
| L orbitofrontal cortex | 110 | 5.20 | 1.000 | -22 | 15 | -22 |
| L superior frontal gyrus, middle frontal gyrus | 586 | 5.17 | 0.290 | -28 | 56 | 18 |
| R fusiform gyrus | 116 | 5.10 | 1.000 | 39 | -57 | -20 |
| R cuneus | 159 | 5.07 | 0.997 | 14 | -102 | 9 |
| R orbitofrontal cortex | 104 | 4.91 | 1.000 | 26 | 20 | -18 |
| R middle temporal gyrus, inferior temporal gyrus, superior temporal gyrus | 715 | 4.87 | 0.158 | 66 | -20 | -21 |
| L rectus gyrus | 117 | 4.77 | 1.000 | -6 | 26 | -18 |
| R superior temporal gyrus | 104 | 4.72 | 1.000 | 64 | -33 | 4 |
| L superior occipital gyrus | 211 | 4.66 | 0.997 | -21 | -86 | 36 |
| L inferior temporal gyrus | 100 | 4.64 | 1.000 | -50 | -60 | -16 |
| R superior occipital gyrus, cuneus | 159 | 4.49 | 0.997 | 22 | -88 | 28 |
| B cuneus | 102 | 4.39 | 1.000 | 4 | -84 | 26 |
| L inferior temporal gyrus | 134 | 4.29 | 0.999 | -45 | -42 | -27 |
| R lingual gyrus, fusiform gyrus | 103 | 4.17 | 1.000 | 20 | -50 | -9 |
| **C3-C4 REM sleep EEG slowing ratio** | | | | | | |
| ***Women*** | | | | | | |
| L superior/middle/inferior temporal gyri, fusiform gyrus, temporal pole, hippocampus, amygdala, insula, putamen, superior/inferior frontal gyri | 4238 | 6.88 | **0.001** | -40 | -10 | -14 |
| R insula, hippocampus, amygdala, parahippocampal gyrus | 1938 | 5.17 | 0.059 | 38 | -4 | -10 |
| Vermis | 111 | 4.98 | 1.000 | 6 | -40 | -21 |
| R insula | 112 | 4.69 | 1.000 | 36 | 22 | 9 |
| R insula, inferior frontal gyrus, superior frontal gyrus | 148 | 4.31 | 0.999 | 26 | 21 | -15 |
| L superior frontal gyrus | 126 | 3.87 | 1.000 | -21 | 58 | 4 |
| L middle frontal gyrus | 101 | 3.75 | 1.000 | -26 | 22 | 46 |
| ***Men*** | | | | | | |
| L precentral gyrus, postcentral gyrus | 521 | 8.93 | 0.537 | -34 | -30 | 60 |
| R superior frontal gyrus | 523 | 8.33 | 0.534 | 20 | -14 | 75 |
| L postcentral gyrus, supramarginal gyrus | 358 | 7.34 | 0.836 | -60 | -16 | 42 |
| R middle/superior frontal gyri | 438 | 6.75 | 0.691 | 33 | 24 | 57 |
| Vermis, L lingual gyrus | 110 | 6.37 | 1.000 | 2 | -72 | -8 |
| R superior/middle frontal gyri | 157 | 5.90 | 0.998 | 24 | 63 | 16 |
| L precentral gyrus, middle frontal gyri | 157 | 5.79 | 0.998 | -51 | -2 | 51 |
| L superior frontal gyrus | 146 | 5.78 | 0.999 | -8 | 38 | 57 |
| L inferior/middle frontal gyri | 282 | 5.73 | 0.940 | -51 | 21 | 34 |
| L middle/superior frontal gyri | 213 | 5.59 | 0.987 | -38 | 50 | 24 |
| L paracentral lobule, precentral gyrus | 104 | 5.26 | 1.000 | -18 | -20 | 76 |
| R supplementary motor area | 111 | 5.06 | 1.000 | 10 | -3 | 51 |
| R middle frontal gyrus | 131 | 5.05 | 1.000 | 38 | 6 | 60 |
| L middle temporal gyrus | 126 | 4.84 | 1.000 | -68 | -28 | 3 |
| **P3-P4 REM sleep EEG slowing ratio** | | | | | | |
| ***Women*** | | | | | | |
| L inferior temporal gyrus, insula, hippocampus, amygdala, parahippocampal gyrus, fusiform, inferior temporal gyrus, temporal pole | 5426 | 6.42 | **<0.001** | -39 | -20 | -22 |
| Vermis | 135 | 5.17 | 0.999 | 3 | -40 | -20 |
| R insula, putamen, hippocampus, amygdala, parahippocampal gyrus | 1719 | 4.90 | 0.097 | 36 | -3 | -10 |
| R thalamus, nucleus accumbens, basal forebrain, caudate nucleus, gyrus rectus, medial orbital gyrus | 703 | 4.23 | 0.664 | 12 | -4 | 12 |
| L superior frontal gyrus | 319 | 4.21 | 0.972 | -26 | 58 | 4 |
| L middle frontal gyrus | 103 | 3.87 | 1.000 | -26 | 24 | 45 |
| ***Men*** | | | | | | |
| L precentral gyrus, postcentral gyrus | 850 | 10.45 | 0.136 | -34 | -30 | 60 |
| R superior frontal gyrus, precentral gyrus, supplementary motor area | 816 | 8.90 | 0.156 | 20 | -15 | 75 |
| L postcentral gyrus, supramarginal gyrus | 276 | 3.83 | 0.936 | -58 | -15 | 45 |
| L precentral gyrus, paracentral lobule, supplementary motor area | 407 | 6.63 | 0.718 | -21 | -22 | 74 |
| R superior frontal gyrus, middle frontal gyrus | 395 | 6.37 | 0.742 | 26 | 63 | 15 |
| L superior frontal gyrus | 187 | 3.55 | 0.993 | -8 | 38 | 57 |
| L superior frontal gyrus | 108 | 3.54 | 1.000 | 27 | 28 | 56 |
| R supplementary motor area | 181 | 3.53 | 0.995 | 9 | -6 | 58 |
| L precuneus, paracentral lobule, superior parietal gyrus | 417 | 6.18 | 0.698 | -4 | -40 | 72 |
| L supplementary motor area, paracentral lobule, middle cingulate gyrus | 148 | 6.11 | 0.999 | -10 | -22 | 50 |
| L middle frontal gyrus, inferior frontal gyrus | 398 | 6.09 | 0.736 | -34 | 26 | 51 |
| L precentral gyrus, middle frontal gyrus | 296 | 5.98 | 0.912 | -50 | -2 | 52 |
| L superior frontal | 130 | 5.92 | 1.000 | -21 | 2 | 70 |
| R middle frontal gyrus, inferior frontal gyrus | 470 | 5.75 | 0.592 | 44 | 28 | 38 |
| R superior parietal gyrus, precuneus | 120 | 5.65 | 1.000 | 15 | -63 | 68 |
| R middle frontal gyrus | 183 | 5.59 | 0.994 | 38 | 6 | 60 |
| L superior frontal gyrus | 322 | 5.32 | 0.874 | -22 | 63 | 9 |
| B lingual gyrus, vermis | 333 | 5.22 | 0.856 | 6 | -60 | -2 |
| L middle temporal gyrus, superior temporal gyrus | 234 | 5.18 | 0.973 | -66 | -30 | 2 |
| L superior frontal gyrus | 100 | 5.15 | 1.000 | -15 | 63 | 20 |
| R superior frontal gyrus | 136 | 5.03 | 0.999 | 9 | 54 | -2 |
| L superior occipital gyrus, middle occipital gyrus | 317 | 4.54 | 0.882 | -21 | -87 | 36 |
| L middle frontal gyrus, orbitofrontal cortex | 114 | 4.50 | 1.000 | -27 | 38 | -12 |
| **O1-O2 REM sleep EEG slowing ratio** | | | | | | |
| ***Women*** | | | | | | |
| R thalamus | 174 | 4.49 | 0.998 | 9 | -6 | 12 |
| L hippocampus, parahippocampal gyrus, amygdala | 387 | 4.34 | 0.950 | -18 | -6 | -16 |
| ***Men*** | | | | | | |
| L postcentral gyrus, precuneus, paracentral lobule | 1745 | 10.65 | **0.004** | -28 | -33 | 69 |
| L precentral gyrus | 370 | 9.44 | 0.779 | 22 | -18 | 75 |
| L superior frontal gyrus | 112 | 7.06 | 1.000 | -8 | 39 | 56 |
| Vermis, B lingual gyrus, R cerebellum | 597 | 6.71 | 0.358 | 4 | -60 | -6 |
| L middle frontal gyrus | 382 | 6.60 | 0.755 | -36 | 24 | 52 |
| R superior frontal gyrus | 286 | 6.52 | 0.919 | 27 | 0 | 68 |
| R superior frontal gyrus | 276 | 6.51 | 0.932 | 27 | 63 | 14 |
| L postcentral gyrus, inferior parietal gyrus | 254 | 6.47 | 0.955 | -58 | -14 | 44 |
| L superior/middle frontal gyrus | 295 | 6.45 | 0.907 | -15 | 20 | 64 |
| L precentral gyrus | 392 | 6.42 | 0.735 | -22 | -21 | 74 |
| R superior parietal gyrus | 196 | 6.11 | 0.990 | 14 | -63 | 66 |
| L middle/superior occipital gyrus | 1521 | 6.08 | **0.009** | -21 | -87 | 34 |
| R superior frontal gyrus | 312 | 5.95 | 0.882 | 26 | 27 | 56 |
| L paracentral lobule | 144 | 5.89 | 0.999 | -9 | -24 | 51 |
| L precentral gyrus | 309 | 5.72 | 0.887 | -36 | 3 | 62 |
| L superior parietal gyrus | 253 | 5.70 | 0.956 | -18 | -62 | 63 |
| L medial orbital gyrus | 115 | 5.41 | 1.000 | 9 | 54 | 0 |
| R precentral gyrus | 110 | 5.30 | 1.000 | 46 | -18 | 62 |
| R middle frontal gyrus | 203 | 5.24 | 0.988 | 39 | 3 | 60 |
| L superior/middle temporal gyrus | 585 | 5.15 | 0.376 | -58 | -3 | -9 |
| R supplementary motor area | 137 | 5.15 | 0.999 | 8 | -6 | 58 |
| R postcentral gyrus | 149 | 5.01 | 0.999 | 14 | -48 | 75 |
| R cerebellum, R fusiform gyrus | 111 | 4.99 | 1.000 | 32 | -36 | -27 |
| R middle/inferior frontal gyri | 330 | 4.92 | 0.853 | 44 | 27 | 44 |
| L inferior temporal gyrus | 132 | 4.90 | 0.999 | -51 | -45 | -27 |
| L inferior/middle frontal gyri | 199 | 4.84 | 0.989 | -27 | 38 | -15 |
| L inferior occipital gyrus, fusiform gyrus | 165 | 4.76 | 0.997 | -51 | -62 | -16 |
| L superior/middle temporal gyri | 271 | 4.65 | 0.938 | -64 | -44 | 18 |
| R middle occipital gyrus, cuneus | 143 | 4.60 | 0.999 | 39 | -88 | 2 |
| R middle/inferior temporal gyri | 230 | 4.55 | 0.974 | 66 | -18 | -22 |
| R cuneus, superior occipital gyrus | 207 | 4.50 | 0.986 | 14 | -90 | 30 |
| L superior/middle frontal gyri | 378 | 4.47 | 0.763 | -15 | 64 | 20 |
| **T3-T4 REM sleep EEG slowing ratio** | | | | | | |
| ***Women*** | | | | | | |
| Vermis | 287 | 7.26 | 0.985 | 8 | -44 | -26 |
| L anterior cingulate cortex, nucleus accumbens, basal forebrain, putamen, caudate nucleus | 387 | 4.41 | 0.953 | -6 | 22 | -6 |
| R caudate nucleus | 173 | 4.37 | 0.998 | 14 | 21 | -8 |
| R superior temporal pole | 105 | 4.06 | 1.000 | 50 | 4 | -12 |
| R putamen | 282 | 3.70 | 0.986 | 30 | 18 | 3 |
| ***Men*** | | | | | | |
| Vermis, B lingual gyrus, R calcarine cortex | 595 | 7.82 | 0.387 | 4 | -63 | -3 |
| L postcentral gyrus, supramarginal gyrus, precentral gyrus | 303 | 7.49 | 0.906 | -57 | -12 | 45 |
| L postcentral gyrus, precentral gyrus | 451 | 6.83 | 0.639 | -28 | -34 | 68 |
| L middle frontal gyrus, inferior frontal gyrus | 468 | 6.70 | 0.606 | -51 | 20 | 36 |
| R middle frontal gyrus, superior frontal gyrus, inferior frontal gyrus | 650 | 6.22 | 0.313 | 42 | 28 | 33 |
| R postcentral gyrus | 106 | 6.18 | 1.000 | 63 | -2 | 28 |
| R superior frontal gyrus, middle frontal gyrus | 559 | 6.17 | 0.442 | 24 | 4 | 62 |
| R precentral gyrus | 117 | 6.13 | 1.000 | 42 | -22 | 64 |
| R superior frontal gyrus | 136 | 5.90 | 0.999 | 16 | 36 | 54 |
| R superior frontal gyrus, middle frontal gyrus | 104 | 5.77 | 1.000 | 26 | 50 | 38 |
| L precentral gyrus, paracentral lobule | 153 | 5.61 | 0.999 | -26 | -24 | 68 |
| R inferior parietal gyrus | 142 | 5.55 | 0.999 | 48 | -45 | 56 |
| L superior frontal gyrus | 137 | 5.48 | 0.999 | -27 | 58 | 12 |
| R precuneus | 122 | 5.45 | 1.000 | 8 | -50 | 72 |
| R precentral gyrus, superior frontal gyrus | 314 | 5.02 | 0.890 | 26 | -20 | 72 |
| R inferior frontal gyrus, insula | 362 | 4.39 | 0.811 | 44 | 26 | 3 |
| **T5-T6 REM sleep EEG slowing ratio** | | | | | | |
| ***Women*** | | | | | | |
| L amygdala, olfactory gyrus, caudate, putamen, rectus gyrus, insula | 3304 | 5.57 | **0.009** | -21 | -6 | -15 |
| Vermis | 152 | 5.06 | 0.999 | 9 | -42 | -28 |
| R caudate, rectus gyrus | 710 | 4.77 | 0.698 | 6 | 14 | -2 |
| R thalamus | 173 | 4.73 | 0.998 | 9 | -6 | 14 |
| R hippocampus, parahippocampal gyrus, amygdala | 616 | 4.51 | 0.784 | 33 | -4 | -18 |
| ***Men*** | | | | | | |
| L postcentral gyrus, precentral gyrus | 313 | 7.95 | 0.854 | -33 | -30 | 58 |
| R superior frontal gyrus, precentral gyrus | 191 | 7.90 | 0.990 | 20 | -15 | 75 |
| R superior frontal gyrus | 221 | 7.15 | 0.974 | 24 | 9 | 64 |
| L postcentral gyrus | 101 | 7.09 | 1.000 | -58 | -15 | 44 |
| Vermis, B cerebellar hemispheres, R lingual gyrus | 315 | 7.03 | 0.850 | 6 | -60 | -8 |
| L superior parietal gyrus | 125 | 6.18 | 1.000 | -22 | -57 | 66 |
| L middle/inferior occipital gyri | 282 | 5.94 | 0.906 | -33 | -93 | 2 |
| R superior frontal gyrus | 110 | 5.54 | 1.000 | 33 | 58 | 14 |

Whole-brain negative voxel-wise multiple regression analyses were performed between REM sleep EEG slowing ratios on F3-F4 (n=12 women, n=10 men), C3-C4 (n=13 women, n=9 men), T3-T4 (n=13 women, n=10 men), T5-T6 (n=13 women, n=9 men), P3-P4 (n=13 women, n=10 men) and O1-O2 (n=13 women, n=10 men) derivations, and FEOBV SUVr maps, in groups stratified by sex. Results are presented at the *P < 0.005* (uncorrected) level combined with a minimum cluster size of *k=100*, controlling for age. Clusters surviving a cluster-level FWE correction are highlighted in bold. For each cluster, coordinates correspond to the statistical peak of the cluster.

Abbreviations: B, bilateral; FWE, family wise error; L, left; MNI, Montreal Neurological Institute; R, right; REM, rapid eye movement.

**Supplementary Table 5. Associations between gray matter volume and REM sleep EEG slowing and FEOBV-PET signal.**

| **Independent variable** | **Dependent variable** | **b (SE)** | **95% CI** | **β** | **t** | **p** | **p_FDR-corr_** |
| --- | --- | --- | --- | --- | --- | --- | --- |
| Total BF volume (mm^3^) | Log(F3-F4 slowing ratio) | 4.01e^-4^ (6.92e^-4^) | -0.001 – 0.002 | 0.13 | 0.58 | 0.57 | 0.57 |
|  | Log(C3-C4 slowing ratio) | 5.00e^-4^ (6.96e^-4^) | -9.63e^-4^ – 0.002 | 0.16 | 0.72 | 0.48 | 0.57 |
|  | Log(P3-P4 slowing ratio) | 7.08e^-4^ (6.41e^-4^) | -6.33e^-4^ – 0.002 | 0.24 | 1.11 | 0.28 | 0.43 |
|  | Log(O1-O2 slowing ratio) | 6.49e^-4^ (4.84e^-4^) | -3.64e^-4^ – 0.002 | 0.26 | 1.34 | 0.20 | 0.40 |
|  | Log(T5-T6 slowing ratio) | 6.36e^-4^ (5.97e^-4^) | -6.19e^-4^ – 0.002 | 0.22 | 1.06 | 0.30 | 0.43 |
|  | Mean SUVr (F3-F4 slowing associated clusters) | -0.001 (8.42e^-4^) | -0.003 – 3.27e^-4^ | -0.34 | -1.70 | 0.11 | 0.37 |
|  | Mean SUVr (C3-C4 slowing associated clusters) | -0.002 (8.51e^-4^) | -0.004 – 6.00e^-5^ | -0.41 | -2.03 | 0.06 | 0.30 |
|  | Mean SUVr (P3-P4 slowing associated clusters) | -0.001 (9.18e^-4^) | -0.003 – 6.63e^-4^ | -0.29 | -1.36 | 0.19 | 0.40 |
|  | Mean SUVr (O1-O2 slowing associated clusters) | -0.002 (8.76e^-4^) | -0.004 – -6.24e^-5^ | -0.40 | -2.16 | 0.04 | 0.30 |
|  | Mean SUVr (T5-T6 slowing associated clusters) | -9.49e^-4^ (0.001) | -0.004 – 0.002 | -0.16 | -0.67 | 0.51 | 0.57 |
| Ch4 volume (mm^3^) | Log(F3-F4 slowing ratio) | 1.17e^-4^ (0.001) | -0.002 – 0.002 | 0.02 | 0.11 | 0.91 | 0.91 |
|  | Log(C3-C4 slowing ratio) | 1.84e^-4^ (0.001) | -0.002 – 0.002 | 0.04 | 0.17 | 0.86 | 0.91 |
|  | Log(P3-P4 slowing ratio) | 6.04e^-4^ (9.92e^-4^) | -0.001 – 0.003 | 0.13 | 0.61 | 0.55 | 0.90 |
|  | Log(O1-O2 slowing ratio) | 5.99e^-4^ (7.55e^-4^) | -9.79e^-4^ – 0.002 | 0.16 | 0.80 | 0.44 | 0.88 |
|  | Log(T5-T6 slowing ratio) | 4.43e^-4^ (9.33e^-4^) | -0.002 – 0.002 | 0.10 | 0.48 | 0.64 | 0.90 |
|  | Mean SUVr (F3-F4 slowing associated clusters) | -0.002 (0.001) | -0.004 – 9.56e^-4^ | -0.27 | -1.35 | 0.19 | 0.63 |
|  | Mean SUVr (C3-C4 slowing associated clusters) | -0.002 (0.001) | -0.005 – 4.87e^-4^ | -0.36 | -1.73 | 0.10 | 0.50 |
|  | Mean SUVr (P3-P4 slowing associated clusters) | -0.001 (0.001) | -0.004 – 0.002 | -0.20 | -0.92 | 0.37 | 0.88 |
|  | Mean SUVr (O1-O2 slowing associated clusters) | -0.003 (0.001) | -0.006 – -3.43e^-5^ | -0.40 | -2.12 | 0.048 | 0.48 |
|  | Mean SUVr (T5-T6 slowing associated clusters) | -7.92e^-4^ (0.002) | -0.005 – 0.004 | -0.08 | -0.36 | 0.72 | 0.90 |
| Ch123 volume (mm^3^) | Log(F3-F4 slowing ratio) | 0.001 (0.001) | -0.001 – 0.004 | 0.21 | 0.96 | 0.35 | 0.39 |
|  | Log(C3-C4 slowing ratio) | 0.001 (0.001) | -0.001 – 0.004 | 0.25 | 1.15 | 0.26 | 0.33 |
|  | Log(P3-P4 slowing ratio) | 0.002 (0.001) | -9.19e^-4^ – 0.004 | 0.27 | 1.32 | 0.20 | 0.29 |
|  | Log(O1-O2 slowing ratio) | 0.001 (8.95e^-4^) | -5.14e^-4^ – 0.003 | 0.29 | 1.52 | 0.15 | 0.29 |
|  | Log(T5-T6 slowing ratio) | 0.002 (0.001) | -7.31e^-4^ – 0.004 | 0.29 | 1.44 | 0.17 | 0.29 |
|  | Mean SUVr (F3-F4 slowing associated clusters) | -0.002 (0.002) | -0.005 – 0.001 | -0.28 | -1.40 | 0.18 | 0.29 |
|  | Mean SUVr (C3-C4 slowing associated clusters) | -0.003 (0.002) | -0.006 – 9.63e^-4^ | -0.32 | -1.52 | 0.15 | 0.29 |
|  | Mean SUVr (P3-P4 slowing associated clusters) | -0.002 (0.002) | -0.006 – 0.001 | -0.28 | -1.35 | 0.19 | 0.29 |
|  | Mean SUVr (O1-O2 slowing associated clusters) | -0.002 (0.002) | -0.006 – 0.001 | -0.26 | -1.33 | 0.20 | 0.29 |
|  | Mean SUVr (T5-T6 slowing associated clusters) | -0.002 (0.003) | -0.008 – 0.003 | -0.19 | -0.81 | 0.43 | 0.43 |

Results of multiple regressions testing the associations between gray matter volume in basal forebrain nuclei (predictors) and (i) REM sleep EEG slowing ratios, and (ii) mean FEOBV-PET SUVr extracted from clusters significantly associated with REM sleep EEG slowing ratios in voxel-wise analyses shown in Figure 2 (outcomes). All models were tested separately and controlled for sex and total intracranial volume. The statistical threshold for significance was set to *P < 0.005* after applying a False Discovery Rate (FDR) correction for multiple comparisons. No results survived this threshold.

Abbreviations: BF, basal forebrain; FEOBV, [^18^F]-Fluoroethoxybenzovesamicol; SE, standard error; SUVr, standard uptake value ratios; REM, rapid eye movement.
